# Photosynthetic Phenomics of Field- and Greenhouse-Grown Amaranths vs. Sensory and Species Delimits

**DOI:** 10.34133/2021/2539380

**Published:** 2021-02-08

**Authors:** S. D. S. S. Sooriyapathirana, L. T. Ranaweera, H. S. M. Jayarathne, T. H. I. Gayathree, P. G. R. G. Rathnayake, S. I. Karunarathne, S. M. N. K. Thilakarathne, R. Salih, C. K. Weebadde, C. P. Weebadde

**Affiliations:** ^1^Department of Molecular Biology and Biotechnology, Faculty of Science, University of Peradeniya, 20400 Peradeniya, Sri Lanka; ^2^PhotosynQ Inc., 325 E Grand River Avenue, Suite 331, East Lansing, MI 48823, USA; ^3^Department of Plant, Soil and Microbial Sciences, College of Agriculture and Natural Resources, Michigan State University, East Lansing 48824, USA

## Abstract

Consumers hesitate to purchase field-grown shoot-tops of amaranths in Sri Lanka, citing the low-cleanliness making growers focus on greenhouse farming. However, the photosynthetic and growth variations in relation to the organoleptic preference of the greenhouse-grown amaranths in comparison to field-grown counterparts have not been studied. Also, the species delimits of the amaranths in Sri Lanka have not been identified, limiting our ability to interpret species-specific production characteristics. Thus, we assessed the common types of amaranths under greenhouse and field conditions. The photosynthesis was measured using a MultispeQ device of the PhotosynQ phenomic platform, which records chlorophyll fluorescence-based parameters. The shoot-tops were harvested and prepared as dishes according to the typical recipe for amaranths in Sri Lanka. The dishes were subjected to an organoleptic assessment for the parameters color, aroma, bitterness, texture, and overall taste. The differences in plant and the shoot-top biomass were also assessed. The markers *atpB-rbcL*, *matk-trnT*, and *ITS* were used to define the species delimits. The field-grown and greenhouse-grown amaranths exhibited species/cultivar-specific photosynthetic variations. The texture and overall taste of the dishes were different among greenhouse and field-grown material. The tasters preferred the texture and the overall taste of the greenhouse-grown shoot-tops. The greenhouse-grown plants also yielded higher shoot-top harvests compared to field-grown counterparts. Out of the tested markers, *ITS* defines the delimits of amaranth species. The higher organoleptic preference, the appreciable yield levels, unique photosynthetic patterns of the greenhouse-grown amaranths, and species definitions provide the much-needed platform for clean shoot-top production guaranteeing the highest end-user trust.

## 1. Introduction

Amaranths are a group of plants that complete their life cycle within a short period. Amaranth belongs to the genus *Amaranthus* of the family Amaranthaceae, a cosmopolitan plant family that completes its life cycle within a short period [1]. One-third of the plants in the genus *Amaranthus* are grown to harvest seeds for consumption as “pseudo” cereal or a leafy vegetable (LV) [2]. The other *Amaranthus* spp. are considered as annual soft weeds. The indigenous people harvest the shoot-tops (i.e., leaves and young stems) to make delicious dishes. Many prefer to pick shoot-tops before the initiation of the terminal inflorescence for highest quality and soft textured tender shoots and leaves for consumption.

Amaranths were historically popular as a nutritious crop as they contain high levels of folic acid, iron, calcium, *β* carotene, and vitamins A, B2, C, and E [3, 4]. However, the main component of the amaranth grain is starch [5]. In addition, amaranth oil can increase the high-density lipoprotein and lower the low-density lipoprotein by 21%-50%. The protein content of the amaranth leaf is about 12% [6], while it is 15% in grains [7]. The protein content of amaranth is similar to the protein level suggested by the Food and Agricultural Organization (FAO) for a balanced diet [8]. Amaranths are also high in vital minerals such as potassium, calcium, magnesium, zinc, iron, manganese, and nickel. Amaranth leaves even contain 7-9% of dietary fibers [4]. Moreover, amaranths are considered as a medicinally important plant species. For example, in traditional medicine, *A*. *spinosus* is used to treat cough and cold, throat and urinary troubles, gastric problems, and broken bones [9, 10]. The amaranth plant extract is used to treat excessive menstruation, diarrhoea, and internal bleeding. Amaranths possess antiviral, hepatoprotective, antiulcer, and antihyperglycemic, antidote, and antinociceptive properties [10–12].

However, *Amaranthus* spp. are considered as a group of underutilized crops despite their immense potential. In Sri Lanka, amaranths grow everywhere, especially in agricultural lands of the mid and low country wet and dry zones. Although the consumption of seeds/grains is widespread in India and South America, amaranths are only consumed as a green/purple LV in Sri Lanka. The application of cattle manure is a routine practice in Sri Lankan vegetable farming. Since the cattle graze or feed on the herbs that appear after the rainy seasons, the manure is filled with nondormant weed seeds, which is dominated by amaranth seeds. Even though amaranths are cultivated to harvest as a LV, the major share comes from the unintentionally grown amaranth plants in the vegetable fields fertilized with cattle manure.

In addition to *Amaranthus* spp., another member of the family Amaranthaceae, cockscomb (CC) (*Celosia cristata* L.), is also a popular green/purple LV in Sri Lanka, which is consumed in the same way as amaranths. CC is also generally considered an amaranth because of the similar taste and same family origin. CC possesses high ornamental value due to its colorful inflorescence and greenish-purple foliage. Amaranths including CC (hereinafter referred to as amaranths) belonged to the traditional diet of the people living in rural areas. However, due to the health concerns and the apparent benefits such as antioxidant and anticancerous properties, amaranths have now found their way as a regular LV all over the country.

However, the preference to consume LV in particular amaranths is often considerably lower because of the lack of cleanliness of the shoot-top bunches available in the market. The consumers always question the hygiene of the materials and the pollution of the sites of harvesting. The consumers often feel petrified by the details of the potential presence of food poisoning microorganisms, nematodes, helminths, protozoans, parasite eggs, and the traces of mammalian excreta on shoot-tops (Pers. Com.). When shoot-top bunches are cleaned and sold in the market, people question the cleanliness of the water that was used to wash the plant material (Pers. Com.).

Therefore, the issue of mistrust on cleanliness can only be addressed if the greenhouse-grown LV is available for consumption. Amaranth produces healthy shoots and leaves under greenhouse conditions within a month since seed germination. It has been reported that the plant biomass accumulation is faster in greenhouses than in the field. The field-grown plants receive more sunlight; however, it causes photoinhibition lowering photosynthetic efficiency and biomass production [13]. Further, the photosynthetic basis behind the higher biomass accumulation in the greenhouse is not known. If grown in greenhouses, amaranth shoot-tops could be harvested weekly with significantly less pest or disease issues. In addition, there is no need for washing before sending greenhouse-grown shoot-top bunches to the market. However, the organoleptic preference of the greenhouse-grown material over the field-grown counterparts has not been assessed and the species delimits of the frequently grown amaranths in Sri Lanka has not been correctly established to date. Thus, we conducted the present study under field and greenhouse conditions to determine the differences in photosynthesis, productivity, and organoleptic preference of frequently consumed amaranths and their species delimits.

## 2. Materials and Methods

### 2.1. Plant Material

The mature seeds were collected from a total of six species/cultivars (two types/cultivars for *A*. *hybridus* subsp. *cruentus* L. and other four are individual species of amaranths) (Table 1). The seeds were germinated and established separately in a greenhouse at Peradeniya, and a field at Nawalapitiya, Sri Lanka, following the standard practices recommended for amaranth cultivation by the Department of Agriculture, Sri Lanka [14]. The names of the species/cultivars and GPS coordinates of the sampling locations are given in Table S1. The field experiments were conducted as raised beds (each bed was 1 m of width and 2 m of length) filled with topsoil added with 80 metric tons per hectare of commercial compost [15]. The plants were arranged in an RCBD layout with 15 replicates (beds). Initially, seeds were sown in lines 30 cm apart. Upon germination, the extra plants were thinned out, leaving 30 cm between plants at 14 days. The basal dressing of urea, triple superphosphate, and muriate of potash was added in the ratio of 64 : 6 : 60 ppm [15]. The greenhouse study was conducted as a pot experiment. The pots (each pot had the dimensions of 30 cm of width, 30 cm of length, and 60 cm of height) were filled with the same soil medium used in the field experiment and basal dressing. The pots were arranged in a CRD layout of 15 reps (pots). Initially, seeds were sown, and a single plant at the center was kept by thinning all the other plants at 14 days. The general crop management practices were applied, and plants were managed until the shoot-tops reached their harvestable stage (just before the initiation of inflorescences).

### 2.2. Variation of the Photosynthesis between Field- and Greenhouse-Grown Plants

The data collection for chlorophyll fluorescence-based photosynthetic traits was carried out at the vegetative stage of plants. The data were collected from 8:00 am to 2:00 pm on multiple days of the growth period using the hand-held MultispeQ device [16]. We randomly sampled ten plants from each species/cultivar separately from field and greenhouse. The photosynthetic traits from the middle and top leaves of the canopy were measured. The photosynthetic measurements were uploaded to the PhotosynQ platform (http://www.photosynq.org). We measured relative chlorophyll content (SPAD), the quantum efficiency for photosystem II photochemistry (*Φ*II), photoprotective nonphotochemical quenching (*Φ*NPQ), basal dissipation of light energy (*Φ*NO), and linear electron flow (LEF) using the MultispeQ. This instrument can also measure the photosynthetically active radiation (PAR), temperature, leaf-environment temperature differential, and humidity variations along with the photosynthetic data. *Φ*II represents the amount of light received by the leaf that goes to the photosystem II and ultimately gets converted to carbohydrates. Therefore, *Φ*II is the best measurement based on chlorophyll fluorescence that indicates the plant's photosynthetic status. On the other hand, *Φ*NPQ is the amount of light dissipated as heat or other energy forms, so that the leaves do not get photodamaged. *Φ*NO is the amount of received light that has not been used by the leaf for photosynthesis or dissipated out. Thereby, *Φ*NO represents potential damage. The summation of *Φ*II, *Φ*NPQ, and *Φ*NO is equal to one. LEF can be considered an indicator of photosynthesis as it reflects the amount of energy that is moving around the chloroplast upon exposure to the light. SPAD indicates the greenness, which is the direct representation of relative chlorophyll content [17].

### 2.3. Assessment of the Organoleptic Preference

All amaranths and CC shoot-tops were harvested a day before the taste panel. The shoot-tops were washed thoroughly and cut into 0.5 cm pieces. The required amount of salt for the taste, 20 g of finely chopped green chili, 50 g of grated coconut, and 40 g of finely chopped onion were added to 1 kg of fresh shoot-top material. The content was mixed in a bowl without squeezing the herbs. Then, 10 ml of coconut oil was heated up in a pan, and the prepared mixture was added and mixed with oil without pressing the content. The pan was closed and kept under slow heat for two mins. Then, the pan was taken out from the heat, and the content was gently mixed and allowed to settle for five mins. The dishes were given to 30 taste panelists within an hour of cooking. The panelists were asked to rank each dish for color, aroma, texture, bitterness, and the overall taste using three levels of preferences (1: least; 2: moderate; 3: highest). Except for the bitterness, 3 means the highest preference, and for the bitterness, 3 was the highest felt bitterness [18].

### 2.4. Plant Growth Differences under Field and Greenhouse Conditions

We measured the number of days to initiate flowering since establishment, the plant height and root length at flowering, fresh and dry weights of single plants, and shoot-tops. We recorded the measurements from ten plants belonging to each amaranth species/cultivars and CC grown under field and greenhouse conditions.

### 2.5. DNA Extraction, PCR, and Sequencing

The immature leaves were collected from five species/cultivars and ground in liquid nitrogen to acquire a fine powder. The powdered samples were stored at -80°C until subsequent extraction steps. The genomic DNA was extracted using the DNeasy® Plant Mini Kit (Qiagen, Solna, Sweden) according to the manufacturer's instructions. The extracted DNA samples were stored at -20°C. The PCR was carried out with 3.5 *μ*l of spermidine (1.34 × 10^−4^ moldm^−3^) for standard universal plant DNA barcoding markers *atpB*-*rbcL*, *matK*-*trnT*, and *ITS* (Table S2). A 15 *μ*l PCR mixture was prepared to contain 2x Go Taq® Green Master Mix (Promega Corporation, Madison, Wisconsin, USA), 1 *μ*l of each forward and reverse primers (0.3 pmol), 3.5 *μ*l of spermidine (1.34 × 10^−4^ moldm^−3^), and 1 *μ*l of DNA template (20 ng/*μ*l). We visualized the PCR products using 2.5% agarose gel electrophoresis (Figure S1), purified the products using the QIAquick® PCR Purification Kit (Qiagen, Hilden, Germany), and cycle-sequenced (3x) using the Genetic Analyzer ABI 3500 (Applied Biosystems®).

### 2.6. Statistical Analysis

The photosynthetic data were subjected to the MIXED procedure in the statistical package SAS 9.4 (SAS Institute, Cary, NC, USA). A multivariate analysis was performed with growth condition, species/cultivar of the plant, leaf position, and the angle, leaf maturity, light intensity, relative humidity, ambient temperature, and leaf temperature differential as covariables in the model. A principal component (PC) analysis followed by a cluster analysis (average linkage and squared Euclidian methods) was conducted for photosynthetic parameters to find the correlations among them and detect the differences among species/cultivars of the plants and the growing conditions (i.e., field vs. greenhouse) using SAS 9.4. The ranked data collected from the taste panel were subjected to the FREQ procedure in SAS 9.4 for the association analysis between each of the taste parameters and the plant species/cultivar. The plant growth data were subjected to GLM procedure, and Tukey mean separation in SAS 9.4. Since the current study focused on the growth differences between field-grown and greenhouse-grown plants, we presented the mean differences only between the growing conditions of each species/cultivar studied.

The sequence polymorphism of *ITS* locus was used to declare the species delimits and to analyze the phylogenetic positions of *Amaranthus* spp. and *C*. *cristata* in Sri Lanka. We included an exemplary dataset reported in [19] which represents most of the extant *Amaranthus* spp. and closely related genera (Table S3). The raw sequence files were initially checked for quality, and the edges were trimmed in MEGA 7 [20]. All the sequences were aligned in MEGA 7 using the Clustal W algorithm [21]. The phylogenies were constructed in both maximum likelihood (ML) and Bayesian frameworks. Since these tree construction methods are associated with the models of the evolution, a selection of the best model was carried out in the software jModelTest [22], in the CIPRES Science Gateway [23]. The ML analysis was carried out in the RAxML [24] platform in the CIPRES supercomputer. We used a rapid bootstrap algorithm with 1000 iterations and a GTRGAMMA model to analyze the dataset. The bootstrap (bs) values and posterior probabilities (PP) were calculated to interpret the rigor of the phylogeny, and the node support was interpreted into the best tree constructed in RAxML. The Bayesian tree was constructed using MrBayes [25] in the CIPRES Science Gateway. A total of four Markov Chain Monte Carlo (MCMC) chains were looped for 20 million generations with a burn-in of 10%. The rigor of the analysis was checked by measuring the effective sample size (ESS) for all the priors.

## 3. Results

### 3.1. Morphological Features

**Figure 1:**
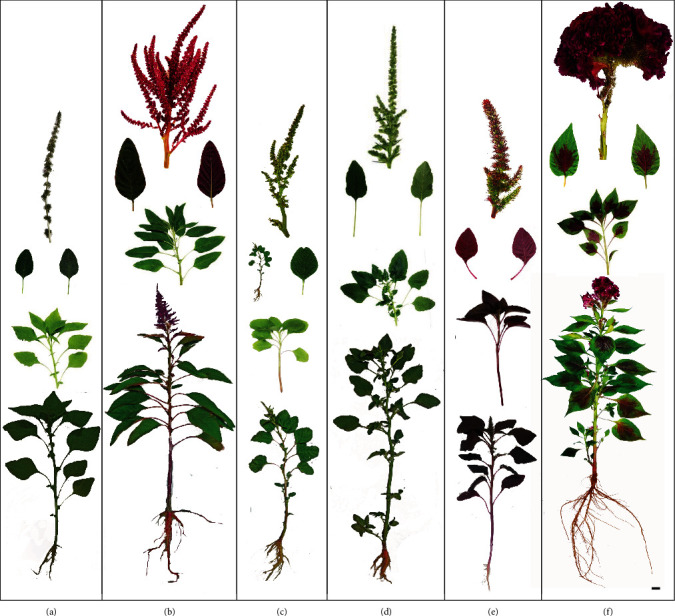
Morphological features of *Amaranthus* spp. and cockscomb. (a) GA-1 (Giant Amaranthus-1 (*A*. *cruentus*)); (b) GA-2 (Giant Amaranthus-2 (*A*. *cruentus*)); (c) GrA (Green Amaranthus (*A*. *viridis*)); (d) TA (Thorny Amaranthus (*A. spinosus*)); (e) RA (Red Amaranthus (*A*. *tricolor*)); (f) CC (cockscomb (*Celocia cristata*)). A mature inflorescence, adaxial and abaxial views of a fully grown leaf, a typical shoot-top harvested for cooking, and a whole plant are shown for each species/cultivar. The scale bar represents 1 cm.

A representative set of the images of the plant species used in the study are given in Figures 1(a)–1(f). *A. hybridus* subsp. *cruentus* has two cultivable forms, GA-1 and GA-2. A pinkish-maroon large axillary or terminally arranged inflorescences can be found in GA-1. A complex thickly branched purple inflorescences are found in GA-2 [26, 27]. *A. viridis* is commonly known as GrA because of the greenish shoot system, which has slender stems [28, 29]. *A. spinosus* is commonly known as TA because of the oppositely arranged strong spines. A. tricolor is popular as RA because of reddish/purplish stems with greenish/purplish leaves. The inflorescences of RA are also red with crimson flowers in globose clusters [28, 30]. *C*. *cristata* is characterized by reddish dense undulating inflorescence that resembles the red color combs on the heads of roosters [31, 32]. The detailed morphological descriptions of these species are provided in Table S4.

### 3.2. Variation of Photosynthesis between Field- and Greenhouse-Grown Plants

The microclimatic conditions in field and greenhouse are summarized in Table 2. The minimum and maximum relative humidity levels were significantly higher in greenhouse conditions. The temperatures were also higher in the greenhouse at least by 2°C. However, the temperature difference between the leaf and the atmosphere was not significantly different among field and greenhouse conditions. Although the minimum PAR is considerably lower under field condition, the maximum and the range of the PAR were significantly higher compared to the greenhouse.

**Figure 2:**
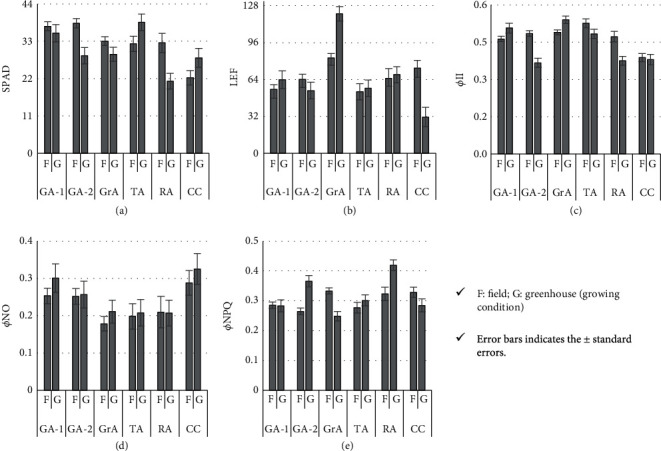
Variation of the photosynthetic parameters of field-grown and greenhouse-grown *Amaranthus* spp. and cockscomb plants.

The photosynthetic parameters SPAD, LEF, *Φ*II, *Φ*NO, and *Φ*NPQ [16] did not show significant differences between the field and greenhouse conditions (Figures 2(a)–2(e)). However, for GA-2 and RA, the field-grown plants possessed a significantly higher amount of chlorophyll as shown by SPAD than the greenhouse-grown plants (Figure 2(a)). Further, amaranth species/cultivars showed specific condition-dependent responses for field and greenhouse conditions. The chlorophyll content, as indicated by the SPAD, was significantly higher in greenhouse-grown CC and TA plants compared to the field-grown plants. The LEF was markedly higher in greenhouse-grown plants in GrA, whereas the field-grown CC plants showed significantly higher LEF than the other field-grown plants (Figure 2(b)). Similarly, the differences between field-grown and greenhouse-grown plants for each species/cultivar for *Φ*II, *Φ*NO, and *Φ*NPQ are shown in Figures 2(c), 2(d), and 2(e), respectively.

**Figure 3:**
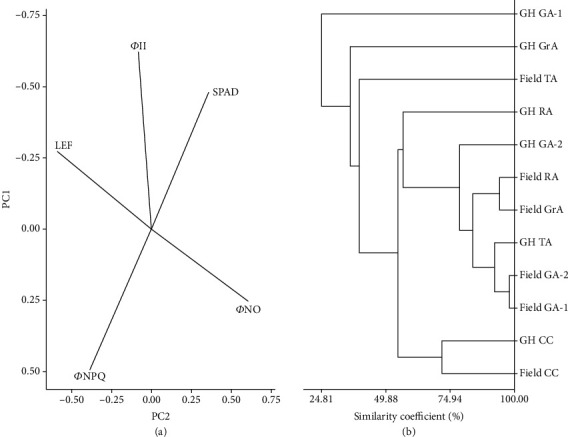
The relationships among the photosynthetic parameters and clustering of amaranth types and CC based on PC analysis. (a) PC loading plot indicating correlations among photosynthetic parameters; (b) dendrogram drawn based on the five PCs calculated for photosynthetic parameters (average linkage and squared Euclidian distance-based) (PC loading status and proportion of variations are given in Table S6).

The smaller or larger significant differences of the photosynthetic parameters among the field-grown and greenhouse-grown plants of each species/cultivars are summarized in Table S5. The PC analysis for the five photosynthetic parameters yielded five PCs, and collectively 79% and 95% of the total variances were explained by the first two and first three principal components, respectively (Table S6). The PC loading status and parametric representation of each PC are also shown in Table S5. As revealed in the PC biplot between two major PCs, it was apparent from the photosynthetic measurements that LEF and *Φ*NO and SPAD and *Φ*NPQ were strongly and negatively correlated with each other separately (Figure 3(a)). Also, *Φ*II and *Φ*NPQ were also negatively correlated with each other. However, *Φ*II and SPAD were highly correlated. The correlations between photosynthetic parameters based on PC biplot are shown in Figure 3(a). When the calculated PC scores were subjected to cluster analysis, the specific responses to growing conditions by the photosynthesis of species/cultivars could be identified. GA-1 and GA-2 demonstrated similar photosynthetic capacities under field conditions; however, under greenhouse conditions, they performed photosynthesis in a drastically different manner. Although the photosynthetic performance of CC plants under greenhouse and field conditions was clustered together, they showed a 34.3% variance. It seems that amaranth species/cultivars have variable adaptations and performances for the field and greenhouse growing conditions (Figure 3(b)).

### 3.3. Consumer Preference

**Figure 4:**
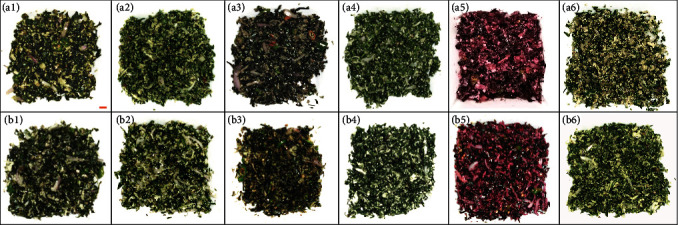
Dishes prepared using shoot-tops for the assessment of organoleptic preference. 1: GA-1 (Giant Amaranthus-1 (*A*. *cruentus*)); 2: GA-2 (Giant Amaranthus-2 (*A*. *cruentus*)); 3: GrA (Green Amaranthus (*A*. *viridis*)); 4: TA (Thorny Amaranthus (*A. spinosus*)); 5: RA (Red Amaranthus (*A*. *tricolor*)); 6: CC (cockscomb (*Celocia cristata*)). (a) Field-grown shoot-tops; (b): greenhouse-grown material. The scale bar shown in orange color indicates 1 cm width of the porcelain dishes.

**Figure 5:**
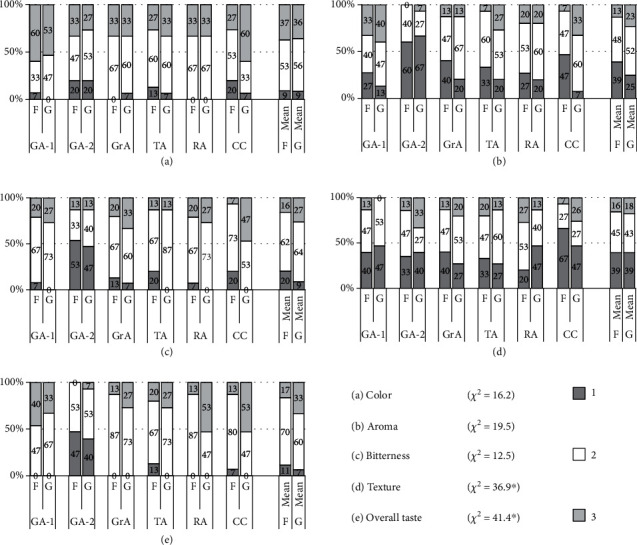
The associations between the taste parameters (a–e) and the type of plant material subjected to taste panel assessment. The chi-square value is given for each association, and ∗ is indicated if the association is significant at *P* < 0.05. *Y*-axis represents the % respondents, and within the stacked bars, relevant % values are shown.

The dishes prepared from the shoot-top material harvested from the field and the greenhouse for the taste panel did not show significant differences (Figure 4). The associations between the species/cultivar of the plant and organoleptic parameters, color, aroma, and bitterness, were not significant (Figures 5(a), 5(b), and 5(c)). It seems that the aroma of the dishes prepared using greenhouse-grown shoot-tops was more attractive than that of field-grown shoot-tops, especially for CC (Figure 5(b)). However, the tasters preferred the color of greenhouse-grown CC more than the field-grown ones (Figure 5(a)). The texture of the greenhouse-grown material was highly preferred compared to field-grown material, as revealed by the significant chi-square value of 36.9 (*P* < 0.05) (Figure 5(d)). The most critical parameter, the overall taste, revealed that for all amaranths, the greenhouse-grown shoot-top material was highly preferred than the field-grown material (Figure 5(e)).

### 3.4. Comparative Growth of the Plants under Field and Greenhouse Conditions

**Figure 6:**
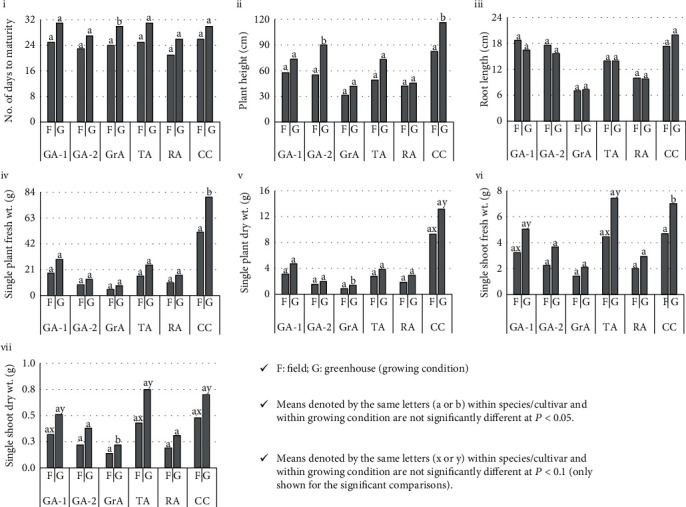
A comparison of the growth of amaranths under field and greenhouse conditions.

We observed the growth of the amaranth plants under field and greenhouse conditions. In the GLM procedure, the species/cultivars and the growing condition had significant effects on all the parameters tested. However, the interaction effect of the species/cultivars and the conditions were not significant for any of the parameters. The field-grown plants matured quickly with limited vegetative growth within three to four weeks and produced the terminal inflorescence, whereas the greenhouse-grown plants showed lush and extensive growth and produced the inflorescence within three to five weeks. However, only GrA showed a significant difference for the mean days to initiate flowering in which field-grown plants initiated flowering six days prior to the greenhouse-grown plants. The mean plant height and the mean root length were significantly different among the species. Although not significantly different in all the comparisons made, the mean plant height was higher in greenhouse-grown plants. However, significantly taller plants were observed in the greenhouse compared to the field only for GA-2 and CC. In general, GA-2 and CC plants showed more prolific growth than the other species/cultivars examined. None of the species/cultivars showed significant differences in mean root length among field-grown and greenhouse-grown plants (*P* > 0.05). The mean fresh and dry weights of the individual plants and the shoot-tops were specific for species/cultivars (*P* < 0.05). The mean fresh weight of the individual plant was significantly higher in greenhouse-grown CC plants compared to the field-grown ones (*P* < 0.05). The mean dry weight of the CC was significantly higher in greenhouse-grown plants (*P* < 0.1). For all the other species/cultivars examined, the field-grown and the greenhouse-grown plants did not show significant differences in mean dry weight within each species/cultivar (*P* > 0.05 or *P* > 0.1). The mean fresh and dry weights were significantly higher in greenhouse-grown plants for CC, GA-1, and TA (Figure 6, i-vii).

### 3.5. Species Delimits and Phylogenetic Analysis

**Figure 7:**
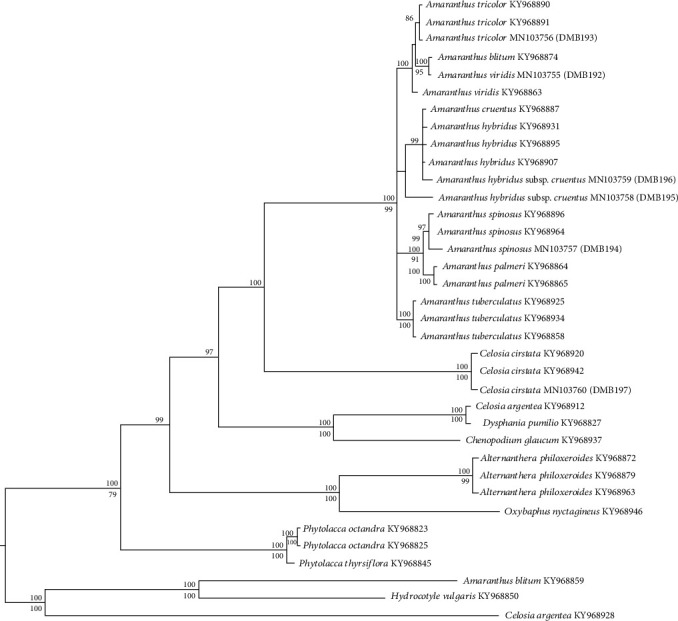
The 50% majority rule consensus tree drawn in the Bayesian framework. The tree shows the phylogenetic positions of *Amaranthus* spp. and *C*. *cristata* plants grown in Sri Lanka based on *ITS* marker polymorphism. The node supports are given above (PP) and below (bs) the node.

The *atpB-rbcL* and *matK-trnT* polymorphisms did not provide sufficient informativeness to identify the species by having higher conservation at sites. However, *ITS* had higher species-specific polymorphic sites enabling us to delimit among species. The phylogenetic trees constructed in both ML and the Bayesian frameworks based on the *ITS* polymorphism had similar topologies. However, the 50% majority rule consensus tree constructed in the Bayesian framework was much resolved than the ML tree. In the phylogeny, all the amaranth species/cultivars considered in the current study nested into a large unigeneric monophyletic clade of *Amaranthus* spp. (*Amaranthus* crown) (posterior probability (PP) = 100, bootstrap (bs) = 99). All the *Amaranthus* spp. studied were correctly positioned at the branches which contained similar species. Moreover, *C. cristata* formed a monophyletic clade (PP = 100, bs = 100), which was nested sister to *Amaranthus* crown (Figure 7).

## 4. Discussion

The amaranth plants show a greater morphological diversity and possess a significant value as ornamental plants (Figure 1). The assessment of the photosynthetic parameters using the MultispeQ device revealed that although the amaranth plants are coming under the same genus, they have diverse adaptations to the prevailing light and other environmental conditions (Figure 2, Table 2). Because of the closed environment and the design, the relative humidity and the temperature are higher in greenhouse conditions than in the field. The temperature difference between the leaf and the environment is maintained at a steady state by the plants as observed (Table 2). The PAR is significantly higher in the field, but when it comes to low light conditions, the greenhouse can maintain a higher PAR than in the field (Table 2). The greenhouse-grown plants are generally taller than field-grown plants, which is in line with our measurements on the less availability of PAR in the greenhouse (Table 2). The increasing plant heights is a well-studied phenomenon under low light conditions [33–35].

The positive correlations between *Φ*II and SPAD indicate that the utilizable amount of light is dependent on the chlorophyll content. The strong correlation between SPAD and *Φ*NPQ reflects that when the leaf contains a higher amount of chlorophyll, the amount of light dissipated as heat/energy is less, implying that the plants utilize a higher proportion of light for biomass assimilation. Similarly, the strong negative correlation between LEF and *Φ*NO indicates that when chloroplasts receive more energy, the amount of light that can cause photodamage gets minimized (Figure 3(a)). However, we observed that the amaranths and CC behave differently under field and greenhouse conditions indicating the species-specific genetic and physiological mechanisms governing the photosynthetic capacity. The assessment of the correlations between chlorophyll fluorescence parameters is a relatively new area in plant sciences. In *Vigna unguiculata* (cowpea or black-eyed pea), the strong negative correlations were observed between *Φ*II and NPQ and *Φ*II and NO when different genotypes were subjected to drought and flooding stress [36]. It is evident from the mean differences of the photosynthetic parameters (Figure 2) and the clustering based on PCs (Figure 3(b)) that the photosynthetic conditions are highly variable among the field and greenhouse conditions. Also, the individual species/cultivars respond in a specific manner to the light and other available growth conditions (Figures 2 and 3(b)).

The dishes prepared using field- and greenhouse-grown shoot-tops are not significantly different in terms of appearance with respect to the species/cultivar of the plants (Figure 4). The association analysis also revealed that the preferred level of the color of the dishes is not significantly associated with the species/cultivars (Figure 5(a)). This similarity is a promising advantage because the consumers would not find any visual differences in dishes prepared from greenhouse-grown material. Similarly, the aroma and the bitterness levels are significantly similar among the field-grown and greenhouse-grown material (Figures 5(b) and 5(c)), indicating that consumers would purchase and consume greenhouse-grown materials without any discrimination. Usually, human beings are so used to consume field-grown plant material. They may think that greenhouse-grown materials would provide a different taste, which could be a challenge for greenhouse LV production. The texture and the overall taste were found to be significantly higher for the greenhouse-grown material indicating that plants grown in fields under natural conditions would produce more hard or fibrous tissues than the greenhouse-grown plants. Also, the greenhouse plants are not subjected to the direct rain, winds, and the impacts due to biotic and abiotic factors, so that their texture is smooth when cooked and the overall taste is also higher (Figures 5(d) and 5(e)). It has been reported that greenhouse-grown fruits and vegetables are softer and possess higher quality [37–39]. As all five *amaranth* species studied possess significant consumer preferences (Figure 5), all these species can be popularized for large-scale LV farming. Currently, CC is only used as an ornamental plant and less popular as a LV. According to the organoleptic preference, the highest palatability was recorded for CC. Therefore, CC should be popularized as a commercial LV in the country. CC is a tropical plant due to its ability to grow well in warmer and drier climates but can be grown in subtropics to a lesser extent. CC is cultivated in several tropical countries, including western tropical Africa for edible shoot-tops [40]. Despite the African origin, CC is now widespread in Asian countries such as Indonesia, China, and India [28]. Moreover, given the predicted climatic changes and growth conditions preferred by CC, this will be a popular LV in warm and dry regions of the equatorial zone in the near future. The ability of CC to grow as a weed with minimum requirements makes it an ideal nutritious LV to be popularized worldwide [41].

Since there is no significant difference among the photosynthetic parameters between field and greenhouse conditions, we paid our attention to the growth rate of the plants under the two conditions. The field-grown plants under natural conditions are smaller and attain the harvesting stage within three weeks. They possess a significantly lower amount of fresh and dry matter compared to those of greenhouse-grown plants (Figure 6). The greenhouse plants attain the harvesting stage within four weeks and exhibit markedly higher growth than the field-grown plants.

Except for the greenhouse-grown GrA plants, we did not detect significant differences in days to initiate flowering under field and greenhouse conditions. However, the data suggest that greenhouse plants take an additional four to five days to initiate flowering, implying that they are more focused on increasing biomass content before the reproduction. The mean root length did not show growing condition-specific significant differences for the species/cultivar probably because of the hardness of the soil in field conditions, and the use of potting medium-filled containers in the greenhouse for planting. Although we observed higher fresh and dry weights for greenhouse-grown plants in each species/cultivar, the significant differences were only detected for CC due to its higher adaptability for the greenhouse conditions. The larger shoot-tops were observed from greenhouse-grown plants for all the studied species/cultivars, indicating that the greenhouse production of amaranths and CC would produce a higher yield than under field conditions. Currently, CC is only used as an ornamental plant and less popular as a LV. According to the organoleptic preference, the highest palatability was recorded for CC. Therefore, CC should be popularized as a commercial LV in the country. We also observed that greenhouse-grown shoot-tops were healthy, attractive, and fresh-looking compared to the shoot-tops harvested from the field-grown plants because the greenhouse-grown plants were not disturbed by pest and disease attacks and extreme physical forces such as direct rainfall, wind, and light. The lower PAR values (Table 2) received by greenhouse-grown plants should have contributed to lower photoinhibition and higher efficiency in photosynthesis leading to efficient biomass accumulation and growth.

The phylogenetic analysis based on a dataset for amaranths [19] and the present study strongly supports the monophyly of amaranths (Figure 7). All the *Amaranthus* species that we studied are consumable and possess an economic potential due to their attractive morphological features and culinary values. Although all the amaranths are not growing extensively as commercial LV, only GA-1 and GrA are currently cultivated as crops and available in the market. The closer phylogenetic relationships of all four amaranth species within the *Amaranthus* genus is evident from the similar floral characteristics of GA-1, GA-2, GrA, RA, and TA (Figures 1(a)–1(e)). Although GA-1 and GA-2 belong to the same species, they are nested in polyphyletic clades within the amaranth crown (Figure 6), which also display a marked morphological difference (Figures 1(a) and 1(b)). Though the GrA examined in this study and the same species listed in the dataset in [19] were nested within the genus *Amaranthus*, their clades are paraphyletic (Figure 6). This result may occur because of the continuous genetic alterations due to adaptive evolutions in diverse locations [42, 43]. Collectively, the current study reports the closer phylogenetic relationships among the members of the genus *Amaranthus* and CC (Figure 7).

## 5. Conclusion

The studied amaranths exhibited a morphological variation and ornamental value due to the presence of attractive foliage and inflorescences. The field-grown and greenhouse-grown plants showed species/cultivar-specific differences in photosynthesis. It was evident from the analysis of the photosynthetic parameters that total chlorophyll content and *Φ*NPQ and LEF and *Φ*NO were negatively correlated to each other. In contrast, chlorophyll content was very highly correlated to *Φ*II. When prepared as LV dishes, field-grown and greenhouse-grown plants did not show any marked differences for each species/cultivar. The tasters preferred the texture and the overall taste of dishes made up of greenhouse-grown shoot-tops compared to that of field-grown materials. This creates an avenue to popularize greenhouse-grown amaranths, among the public to provide clean LV to safeguard the public health. In general, greenhouse-grown amaranths produce higher shoot-top yields compared to field-grown plants. The phylogenetic analysis revealed that out of *atpB-rbcL*, *matk-trnT*, and *ITS* markers, *ITS* is only capable of delimiting the amaranth species, which enables the authentication of the species origin of amaranths in the supply chains and the markets of LV and herbal medicine.

## Figures and Tables

**Table 1:** The details of the species studied.

Botanical name	Common name	Abbreviation
*A. hybridus* subsp. *cruentus* L.	Giant Amanathus-1	GA-1
*A. hybridus* subsp. *cruentus* L.	Giant Amaranthus-2	GA-2
*A. viridis* L.	Green Amaranthus	GrA
*A. spinosus* L.	Thorny Amaranthus	TA
*A. tricolor* L.	Red Amaranthus	RA
*C*. *cristata* L.	Cockscomb	CC

**Table 2:** Summary of the microclimatic conditions in field and greenhouse.

Growing condition	Statistics	Relative humidity (%)	Temp. (°C)	Leaf temperature difference (°C)	PAR
Field	Min	19.29	28.27	-12.57	10.45
	Max	57.02	39.58	1.34	3342.75
GH	Min	34.36	29.12	-12.15	25.04
	Max	66.12	41.27	0.54	2982.82

## Data Availability

The DNA sequence data were submitted to NCBI-GenBank under the accession numbers MN104855-MN104860 (*atpB-rbcL*), MN104861-MN104866 (*matK-trnT*), and MN103755-MN103760 (*ITS*). Also, the data collection protocol, along with the measurements as a project, was deposited in the PhotosynQ web portal for free access (http://www.photosynq.org/projects/amaranthus). The raw data for plant growth comparisons and organoleptic preference are available upon request to the authors.

## Supplementary Materials

Supplementary Materials Table S1: sampling locations. Table S2: the details of the DNA barcoding markers and PCR. Table S3: the DNA sequences used for the phylogenetic analysis. Table S4: morphological descriptions of Amaranthus spp. and cockscomb. Table S5: summary of the differences of photosynthetic parameters. Table S6: summary of PC analysis for photosynthetic parameters. Figure S1: PCR bands obtained for the three plant DNA barcoding markers used. A: *atpB-rbcL*; B: *matK-trnT*; C: *ITS 1-4*. L: 100 bp DNA ladder; GA-1: Giant Amaranthus-1 (*A. cruentus*); GA-2: Giant Amaranthus-2 (*A. cruentus*); GrA: Green Amaranthus (*A. viridis*); TA: Thorny Amaranthus (*A. spinosus*); CC: cockscomb (*Celocia cristata*); RA: Red Amaranthus (*A. tricolor*); +: positive control (rice DNA); -: negative control (no template in PCR). The sizes of the two key bands are shown for each marker.

## References

[1] Xu F., Sun M. (2001). Comparative Analysis of Phylogenetic Relationships of Grain Amaranths and Their Wild Relatives (*Amaranthus*; Amaranthaceae) Using Internal Transcribed Spacer, Amplified Fragment Length Polymorphism, and Double-Primer Fluorescent Intersimple Sequence Repeat Markers. *Molecular Phylogenetic Evolution*.

[2] Grubben G. J. H., Grubben G. J. H., Denton O. A. (2004). Amaranthus cruentus. *Plant resources of tropical Africa 2. Vegetables*.

[3] Priya V. P., Celine V. A., Gokulapalan C., Rajamony L. (2007). Screening amaranth genotypes (*Amaranthus* spp.) for yield and resistance to leaf blight caused by *Rhizoctonia solani* Kuhn. *Plant Genetic Research Newsletter*.

[4] Shukla S., Bhargava A., Chatterjee A., Srivastava A., Singh S. P. (2006). Genotypic variability in vegetable amaranth (*Amaranthus tricolor* L.) for foliage yield and its contributing traits over successive cuttings and years. *Euphytica*.

[5] Wu H. X., Corke H. (1999). Genetic diversity in physical properties of starch from a world collection *ofAmaranthus*. *Cereal Chemistry*.

[6] Shukla S., Pandey V., Pachauri G., Dixit B. S., Banerji R., Singh S. P. (2003). Nutritional contents of different foliage cuttings of vegetable amaranth. *Plant Foods for Human Nutrition*.

[7] Rodas B., Bressani R. (2009). The oil, fatty acid and squalene content of varieties of raw and processed amaranth grain. *Archivos Latinoamericanos De Nutricion*.

[8] FAO (1973). Energy and protein requirements. *Food and Agricultural Organization Nutrition Meetings Report Series, no. 52*.

[9] Baral M., Datta A., Chakraborty S., Chakraborty P. (2011). Pharmacognostic studies on stem and leaves of *Amaranthus spinosus* linn. *International Journal of Applied Biology and Pharmaceutical Technology*.

[10] Rastogi A., Shukla S. (2013). Amaranth: a new millennium crop of nutraceutical values. *Critical Reviews in Food Science and Nutrition*.

[11] Al-Dosari M. S. (2012). The effectiveness of ethanolic extract of *Amaranthus tricolor* L.: a natural hepatoprotective agent. *The American Journal of Chinese Medicine*.

[12] Devaraj V. C., Krishna B. G. (2011). Gastric antisecretory and cytoprotective effects of leaf extracts of *Amaranthus tricolor* Linn. in rats. *Journal of Chinese Integrative Medicine*.

[13] Ibrahim M. H., Jaafar H. Z. E. (2011). Photosynthetic capacity, photochemical efficiency and chlorophyll content of three varieties of *Labisia pumila* Benth. exposed to open field and greenhouse growing conditions. *Acta Physiologiae Plantarum*.

[14] Department of Agriculture, Sri Lanka, (DOA) Released and recommended new crop varieties by the varietal release committee of the Department of Agriculture Sri Lanka Sri Lanka. https://www.doa.gov.lk/images/Newvaraties/VRCbook2013.pdf.

[15] Wu H., Sun M., Yue S. (2000). Field evaluation of an *Amaranthus* genetic resource collection in China. *Genetic Resources and Crop Evolution*.

[16] Kuhlgert S., Austic G., Zegarac R. (2016). MultispeQ Beta: a tool for large-scale plant phenotyping connected to the open PhotosynQ network. *Royal Society Open Science*.

[17] Kramer D. M., Johnson G., Kiirats O., Edwards G. E. (2004). New fluorescence parameters for the determination of QARedox state and excitation energy fluxes. *Photosynthesis Research*.

[18] Kumari S. A. S. M., Nakandala N. D. U. S., Nawanjana P. W. I. (2019). The establishment of the species-delimits and varietal-identities of the cultivated germplasm of *Luffa acutangula* and *Luffa aegyptiaca* in Sri Lanka using morphometric, organoleptic and phylogenetic approaches. *PLoS One*.

[19] Xu S. Z., Li Z. Y., Jin X. H. (2018). DNA barcoding of invasive plants in China: a resource for identifying invasive plants. *Molecular Ecology Resources*.

[20] Kumar S., Stecher G., Tamura K. (2016). MEGA7: Molecular Evolutionary Genetics Analysis version 7.0 for bigger datasets. *Molecular Biology and Evolution*.

[21] Thompson J. D., Higgins D. G., Gibson T. J. (1994). CLUSTAL W: improving the sensitivity of progressive multiple sequence alignment through sequence weighting, position-specific gap penalties and weight matrix choice. *Nucleic Acids Research*.

[22] Posada D. (2008). jModelTest: phylogenetic model averaging. *Molecular Biology and Evolution*.

[23] Miller M. A., Pfeiffer W., Schwartz T. Creating the CIPRES Science Gateway for inference of large phylogenetic trees.

[24] Stamatakis A. (2006). RAxML-VI-HPC: maximum likelihood-based phylogenetic analyses with thousands of taxa and mixed models. *Bioinformatics*.

[25] Huelsenbeck J. P., Ronquist F. (2001). MRBAYES: Bayesian inference of phylogenetic trees. *Bioinformatics*.

[26] BSBI List Botanical Society of Britain and Ireland, Archived from the original (xls) on 2015-01-25. https://www.webcitation.org/6VqJ46atN?url=.

[27] Plants for a future. http://pfaf.org/user/plant.aspx?.

[28] Grubben G. J. H., Denton O. A. (2004). *Plant Resources of Tropical Africa 2. Vegetables*.

[29] Yoshitaka T., Nguyen V. K. (2007). *Edible Wild Plants of Vietnam: The Bountiful Garden*.

[30] Korea National Arboretum (2015). *English names for Korean native plants via Korea Forest Service*.

[31] Flora of China (2014). Celosia cristata. *Missouri Botanical Garden, St. Louis,*.

[32] Pritzel G. A., Jessen C. (1882). The German folk name of the plants. *New contribution to the German language treasure*.

[33] Rijkers T., Pons T. L., Bongers F. (2000). The effect of tree height and light availability on photosynthetic leaf traits of four neotropical species differing in shade tolerance. *Functional Ecology*.

[34] Nagashima H., Hikosaka K. (2011). Plants in a crowded stand regulate their height growth so as to maintain similar heights to neighbours even when they have potential advantages in height growth. *Annals of Botany*.

[35] Texas A&M- Agrilife Extension. https://www.aggie-horticulture.tamu.edu/ornamental/a-reference-guide-to-plant-care-handling-and-merchandising/light-temperature-and-humidity/.

[36] Osei-Bonsu I., Hoh D., Cruz J. Variation in chlorophyll fluorescence-derived photosynthetic parameters and SPAD of cowpea genotypes subjected to drought and flooding stress at the pod filling stage. http://bit.ly/35WtlVy.

[37] Greenhouse Organic Pest Management What you need to know about eating safe fruits and vegetables. https://www.growingspaces.com/what-you-need-to-know-about-eating-safe-fruits-and-vegetables/.

[38] npr Vegetables under glass: greenhouses could bring us better winter produce. https://www.npr.org/sections/thesalt/2015/12/08/458774088/veggies-under-glass-greenhouses-could-bring-us-better-winter-produce/.

[39] USDA What’s the dirt on greenhouse organic?. https://www.produceretailer.com/article/news-article/whats-dirt-greenhouse-organic/.

[40] Tindall H. D. (1983). Vegetables in the Tropics.

[41] National Research Council (2006). *Lost Crops of Africa, Volume II: Vegetables*.

[42] Gottlieb L. D. (1984). Genetics and morphological evolution in plants. *The American Naturalist*.

[43] Van Tienderen P. H. (1991). Evolution of generalists and Specialist in spatially heterogeneous environments. *Evolution*.

