# Supplementary material for: Photosynthetic Phenomics of Field- and Greenhouse-Grown Amaranths vs. Sensory and Species Delimits
Source: Plant Phenomics. 2021 Feb 8;2021:2539380. doi: 10.34133/2021/2539380 (PMC7888303; doi:10.34133/2021/2539380)
Supplement: Supplementary Materials — Table S1: sampling locations. Table S2: the details of the DNA barcoding markers and PCR. Table S3: the DNA sequences used for the phylogenetic analysis. Table S4: morphological descriptions of Amaranthus spp. and cockscomb. Table S5: summary of the differences of photosynthetic parameters. Table S6: summary of PC analysis for photosynthetic parameters. Figure S1: PCR bands obtained for the three plant DNA barcoding markers used. A: atpB-rbcL; B: matK-trnT; C: ITS 1-4. L: 100 bp DNA ladder; GA-1: Giant Amaranthus-1 (A. cruentus); GA-2: Giant Amaranthus-2 (A. cruentus); GrA: Green Amaranthus (A. viridis); TA: Thorny Amaranthus (A. spinosus); CC: cockscomb (Celocia cristata); RA: Red Amaranthus (A. tricolor); +: positive control (rice DNA); -: negative control (no template in PCR). The sizes of the two key bands are shown for each marker. [file 2539380.f1.pdf]

Supplementary Materials

Photosynthetic phenomics of field and greenhouse grown amaranths vs. sensory and species delimits

S. D. S. S. Sooriyapathirana[1,2 *], L. T. Ranaweera[1], H. S. M. Jayarathne[1], T. H. I. Gayathree[1], P. G. R. G. Rathnayake[1], S. I. Karunarathne[1], S. M. N. K. Thilakarathne[1], R. Salih[1], C. K. Weebadde[3], C. P. Weebadde[2]

[1]Department of Molecular Biology and Biotechnology, Faculty of Science, University of Peradeniya, 20400, Peradeniya, Sri Lanka

[2]PhotosynQ Inc., 325 E Grand River Avenue, Suite 331, East Lansing, MI 48823, USA.

[3]Department of Plant, Soil and Microbial Sciences, College of Agriculture and Natural Resources, Michigan State University, East Lansing, 48824, USA

*Corresponding author - Email: sunethuop@gmail.com

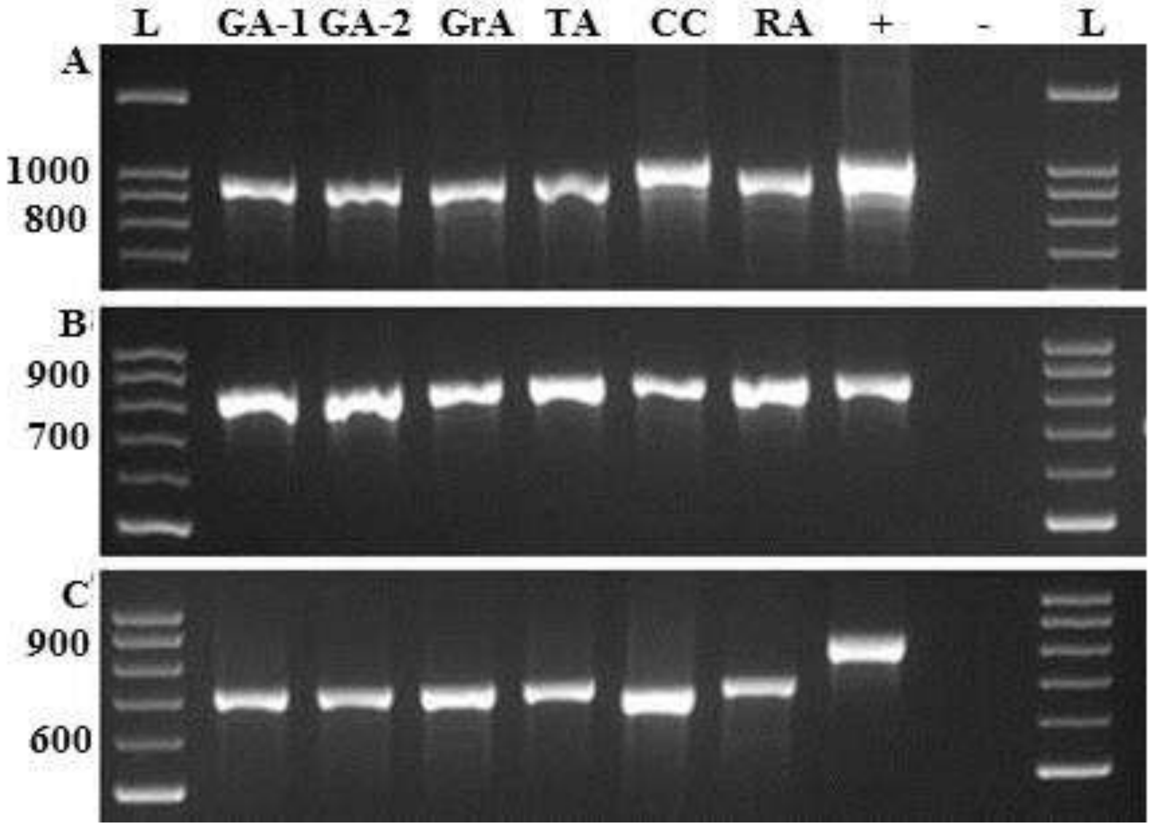

Figure S1. PCR bands obtained for the three plant DNA barcoding markers used. A: atpB- rbcL; B: matK-trnT; C: ITS 1-4. L: 100bp DNA Ladder; GA-1: Giant Amaranthus-1 [A. cruentus]; GA-2: Giant Amaranthus-2 [A. cruentus]; GrA: Green Amaranthus [A. viridis]; TA: Thorny Amaranthus [A. spinosus]; CC: Cockscomb [Celocia cristata]; RA: Red Amaranthus [A. tricolor], + : Positive control [rice DNA]; - : Negative control [no template in PCR]. The sizes of the two key bands are shown for each marker.

S1 Table. Sampling locations

Botanical name	Common name	Abbreviation	Location and GPS Coordinates
A. hybridus subsp. *cruentus* L.	Giant Amanathus-1	GA-1	*Peradeniya* (N 7° 16’ 11.51”; E 80° 35’ 37.80”)
A. hybridus subsp. *cruentus* L.	Giant Amaranthus-2	GA-2	*Balalla* (N 7° 48’ 25.53”; E 80° 15’ 15.37”)
A. viridis L.	Green Amaranthus	GrA	*Nawalapitiya* (N 7° 2’ 40.92”; E 80° 30’ 57.82”)
A. spinosus L.	Throrny Amaranthus	TA	*Gampola* (N 7° 10’ 23.75”; E 80° 33’ 37.84”)
A. tricolor L.	Red Amaranthus	RA	*Weligalla* (N 7° 11’ 19.96”; E 80° 35’ 29.05”)
C. cristata L.	Cockscomb	CC	*Gampola* (N 7° 10’ 23.75”; E 80° 33’ 37.84”)

S2 Table. The details of the DNA barcoding markers and PCR

**Marker**	**Sequence (5'→3')**	**PCR Profile**										**References**
		**Initial denaturation**		**Denaturation**		**Primer Annealing**		**Initial extension**		**Final extension**		
		**T[x]**	**Time**	**T**	**Time**	**T**	**Time**	**T**	**Time**	**T**	**Time**	
atpB-rbcL spacer	P_F: GAAGTAGTAGGATTGATTCTC<br>P_R: TACAGTTGTCCATGTACCAG	94	4 min	94	30 sec	45	30 sec	72	2 min	72	5 min	[1]
matK-trnT spacer	P_F: GCATAAATATAYTCCYGAAARATAAGTGG<br>P_R: TGGGTTGCTAACTCAATGG	95	1.5 min	95	30 sec	48	1 min	68	2 mins	68	20 mins	[2]
ITS1-4	P_F: TCCGTAGGTGAACCTTGCGG<br>P_R: TCCTCCGCTTATTGATATGC	95	3 min	95	1 min	55	1 min	72	1.5 min	72	4 min	[3]

[x]Temperatures (T) are given in ºC.

References

[1] S. B. Hoot and W. C. Taylor, "The utility of nuclear *ITS*, a LEAFY homolog intron, and chloroplast *atpB-rbcL* spacer region data in phylogenetic analyses and species delimitation in Isoetes," *American Fern Journal*, vol. 91, no. 3, pp. 166-177, 2001.

[2] S. Wicke and D. Quandt, "Universal primers for the amplification of the plastid *trnK*/*matK* region in land plants," *Analesdel Jardín Botánico de Madrid*, vol. 66 no. 2, pp. 285-288, 2009.

[3] T. J. White, T. Bruns, S. Lee and J. W. Taylor, "Amplification and direct sequencing of fungal ribosomalRNA genes for phylogenetics," in *PCR Protocols: A Guide to Methods and Applications*, M. A. Innis, D. H. Gelfand, J. J. Sninsky and T.J. White, Eds., pp. 315-322, Academic Press, Inc, New York, 1990.

S3 Table. The DNA sequences used for the phylogenetic analysis

Species	Families	Location	Voucher number	Genbank accesion number			References
				ITS	***atpB-rbcL***	***matK-trnT***	
Oxybaphus nyctagineus	Nyctaginaceae	Beijing, Haidian	13524	KY968946	Not assessed	Not assessed	Xu *et al*., (2017)
Chenopodium glaucum	Chenopodiaceae	Jiangsu, Jiangyin	13506	KY968937	Not assessed	Not assessed	
Dysphania pumilio	Chenopodiaceae	Jiangsu, Jiangyin	13316	KY968827	Not assessed	Not assessed	
Alternanthera philoxeroides	Amaranthaceae	Hunan, Changsha	201509177	KY968879	Not assessed	Not assessed	
Alternanthera philoxeroides	Amaranthaceae	Hunan, Changsha	201509153	KY968872	Not assessed	Not assessed	
Amaranthus blitum	Amaranthaceae	Hunan, Changsha	201509155	KY968874	Not assessed	Not assessed	
Amaranthus blitum	Amaranthaceae	Liaoning, Dalian	2015054	KY968859	Not assessed	Not assessed	
Amaranthus cruentus	Amaranthaceae	Hunan, Zhuzhou	201509205	KY968887	Not assessed	Not assessed	
Amaranthus hybridus	Amaranthaceae	Hunan, Leiyang	201509228	KY968895	Not assessed	Not assessed	
Amaranthus hybridus	Amaranthaceae	Jiangsu, Zhenjiang	13369	KY968907	Not assessed	Not assessed	
Amaranthus hybridus	Amaranthaceae	Jiangsu, Jiangyin	13489	KY968931	Not assessed	Not assessed	
Amaranthus palmeri	Amaranthaceae	Hunan, Changsha	201509140	KY968864	Not assessed	Not assessed	
Amaranthus palmeri	Amaranthaceae	Hunan, Changsha	201509141	KY968865	Not assessed	Not assessed	
Amaranthus spinosus	Amaranthaceae	Hunan, Leiyang	201509229	KY968896	Not assessed	Not assessed	
Amaranthus tricolor	Amaranthaceae	Hunan, Zhuzhou	201509212	KY968890	Not assessed	Not assessed	
Amaranthus tricolor	Amaranthaceae	Hunan, Zhuzhou	201509213	KY968891	Not assessed	Not assessed	
Amaranthus viridis	Amaranthaceae	Hunan, Changsha	201509139	KY968863	Not assessed	Not assessed	
Celosia argentea	Amaranthaceae	Jiangsu, Taizhou	13398	KY968912	Not assessed	Not assessed	
Celosia argentea	Amaranthaceae	Jiangsu, Yangzhou	13482	KY968928	Not assessed	Not assessed	
Celosia cristata	Amaranthaceae	Jiangsu, Taizhou	13442	KY968920	Not assessed	Not assessed	
Celosia cristata	Amaranthaceae	Beijing, Haidian	13519	KY968942	Not assessed	Not assessed	
Hydrocotyle vulgaris	Umbelliferae	Yunnan, Kunming	201507071	KY968850	Not assessed	Not assessed	
Amaranthus tuberculatus	Amaranthaceae	Liaoning, Dalian	2015052	KY968858	Not assessed	Not assessed	
Amaranthus tuberculatus	Amaranthaceae	Jiangsu, Lianyungang	13461	KY968925	Not assessed	Not assessed	
Amaranthus tuberculatus	Amaranthaceae	Jiangsu, Jiangyin	13495	KY968934	Not assessed	Not assessed	
Phytolacca octandra	Phytolaccaceae	Jiangsu, Jiangyin	13305	KY968823	Not assessed	Not assessed	
Phytolacca octandra	Phytolaccaceae	Jiangsu, Jiangyin	13311	KY968825	Not assessed	Not assessed	
Phytolacca thyrsiflora	Phytolaccaceae	Yunnan, Kunming	201507058	KY968845	Not assessed	Not assessed	
Alternanthera philoxeroides	Amaranthaceae	Hunan, border inspection	X12	KY968963	Not assessed	Not assessed	
Amaranthus spinosus	Amaranthaceae	Jiangsu, border inspection	X13	KY968964	Not assessed	Not assessed	
Amaranthus viridis	Amaranthaceae	Sri Lanka	DMB192	MN103755	MN104855	MN104861	This study
Amaranthus tricolor	Amaranthaceae	Sri Lanka	DMB193	MN103756	MN104856	MN104862	
Amaranthus spinosus	Amaranthaceae	Sri Lanka	DMB194	MN103757	MN104857	MN104863	
Amaranthus hybridus subsp. *Cruentus*	Amaranthaceae	Sri Lanka	DMB195	MN103758	MN104858	MN104864	
Amaranthus hybridus subsp. *Cruentus*	Amaranthaceae	Sri Lanka	DMB196	MN103759	MN104859	MN104865	
Celosia cristata	Amaranthaceae	Sri Lanka	DMB197	MN103760	MN104860	MN104866	

S4 Table. Morphological descriptions of *Amaranthus* spp. and cockscomb

Vernacular names	Botanical name	Botanical description
Giant Amaranthus-1 (GA-1), Mexican grain amaranth, *Yodha Thampala*-1	*A. hybridus* subsp. *cruentus*	***Stems:*** greenish or reddish-purple, erect, stout, glabrous, cylindrical stems with ridges reaching up to about 150 cm – 180 cm in height. ***Leaves:*** greenish-purple, simple, long petiolated, broadly lanceolate to rhombic ovate shaped, length varies from 15 cm – 20 cm, width varies from 4 cm – 5 cm. ***Inflorescence:*** pink to maroon, large, complex inflorescence consisting of numerous agglomerated cymes arranged in axillary and terminal racemes and spikes growing up to about 40 cm in length with many lateral, perpendicular thin branches (Figure 1(a)). ***Flowers:*** reddish, subsessile flowers arranged in a large complex inflorescence. ***Fruits:*** dry dehiscent fruit capsule and ovoid to ellipsoid in shape, compressed brownish-black seeds [1, 2].
Giant Amaranth-2 (GA-2), Purple Amaranth, Prince-of-Wales feather, *Yodha Thampala*-2	*A. hybridus* subsp. *cruentus*	***Stems:*** greenish, erect, cylindrical, stout and about 100 cm – 150 cm in height. ***Leaves:*** greenish, simple, long petiolated, entire margined, minutely emarginated leaf apex, broadly lanceolate to rhombic ovate shaped leaves which vary from 15 cm – 18 cm in length and 4 cm – 8 cm in width. ***Inflorescence:*** large, purple-greenish, complex inflorescence arranged into spikes at thick branches (Figure 1(b)). ***Flowers:*** small, greenish, flowers arranged in spikes. ***Fruits:*** dehiscent fruit capsules and ovoid in shape, ellipsoid brownish seeds [1, 2].
Green Amaranth (GrA), Slender amaranth, *Kola Thampala*	*A. viridis*	***Stems:*** greenish, erect, slender, branched, glabrous, rounded stems with ridges which reach up to about 6 cm – 80 cm in height. ***Leaves:*** light greenish, simple, minutely emarginated leaf apex, obtuse base, entire to sinuate margined, deeply veined, long petiolated, ovate to lanceolate shaped, leaves varied from 7 cm – 9 cm in length and 4 cm – 5 cm in width. ***Inflorescence:*** greenish, slender, branched axillary to the terminal paniculate spikes or in the lower part of the stem as dense axillary clusters, 2 cm – 12 cm in length and 2 mm – 5 mm in width (Figure 1(c)). ***Flowers:*** small, greenish male and female flowers arranged in a radial pattern along the spike/inflorescence. ***Fruits:*** wrinkled, indehiscent, small, and brownish fruits with glossy blackish, sub globose, slightly compressed, acute margined seeds [3, 4].
Thorny Amaranthus (TA), Spiny amaranth, Spiny pigweed, Prickly amaranth, *Katu Thampala*	*A. spinosus*	***Stems:*** greenish or reddish-purple, erect or proximally ascending, heavily-branched, hard, obtusely angular, bushy, simple, stems growing up to about 60 cm – 150 cm in height, possess nodes comprised of oppositely arranged strong spines (modified bracts) of about 1.5 cm – 2.5 cm in length observed at the leaf axil. ***Leaves:*** dark greenish, simple, alternate, glabrous or sparse hairs below the main vein, long petiolated, entire margined, minutely emarginated leaf apex, elliptic to lanceolate-oblong shaped leaves. ***Inflorescence:*** greenish arranged into spikes at the thick branches (Figure 1(d)). ***Flowers:*** tiny greenish flowers; unisexual grouped in clusters in the axils of the leaves and branched terminal spikes. ***Fruits:*** dehiscent fruit capsules and ovoid in shaped fruits having 5 mm – 10 mm diameters with blackish, smooth, lens-shaped seeds having a thin margin [5-7].
Red amaranth (RA), *Rathu thampala*, Bireum, Tandaljo, Tandalja bhaji	*A. tricolor*	***Stems:*** reddish to purplish, stout, much-branched, glabrous stems of about 100 cm – 125 cm in height. ***Leaves:*** greenish or purplish suffused, long petiolated, minutely emarginated apex, obtuse base, entire to sinuate margined, rhomboid-ovate or elliptic to lanceolate- oblong-shaped varied from 7 cm – 12 cm in length and 4 cm – 5 cm in width. ***Inflorescence:*** reddish inflorescence with axillary globose clusters of which upper clusters form terminal spikes, with male and female flowers intermixed (Figure 1(e)). ***Flowers:*** crimson flowers in globose clusters. ***Fruits:*** ovoid-shaped fruits 1.5 mm in length, and 1 mm – 1.5 mm in diameter with shiny brownish, smooth, lenticular seeds [3, 8].
Cockscomb (CC), *Kukulkaramal*	*C. cristata*	***Stems:*** greenish, erect, herbaceous stems which grow up to about 30 cm – 100 cm in height. ***Leaves:*** light greenish with some maroon patches, simple, entire margined, rhombic ovate shaped, vary from 7 cm – 9 cm in length and 4 cm – 5 cm in width. ***Inflorescence:*** reddish dense undulating inflorescence that resembles the red color combs on the heads of roosters (Figure 1(f)). ***Flowers:*** brightly pinkish flowers which exhibited three parts; spikes, plumes, and crest. **Fruits**: thin, ovoid to almost globular 1.6 mm – 2.5 mm long fruit capsules and compressed, shiny blackish seeds [9, 10].

References of S4 Table

[1] BSBI List, “Botanical Society of Britain and Ireland, Archived from the original (xls) on 2015-01-25,” 2007, https://www.webcitation.org/6VqJ46atN?url=, http://www.bsbi.org.uk/BSBIList2007.xls.

[2] “Plants for a future,” 2019, https: //pfaf. org/user /plant. aspx? Latin Name = *Amaranthus+hypochondriacus*.

[3] G. J. H. Grubben and O. A. Denton, Plant Resources of Tropical Africa 2. Vegetables, Backhuys Publishers, Wageningen, 2004.

[4] T. Yoshitaka and V. K. Nguyen, *Edible wild plants of Vietnam: The Bountiful Garden*, pp. 24, Orchid Press, Thailand, 2007.

[5] CABI, “Invasive Species Compendium,” 2019, https: //www. cabi.org/ isc/datash eet/4653.

[6] ITIS Report, “Detailed coverage of invasive species threatening livelihoods and the environment worldwide- Amaranthaceae and Phytolaccaceae of North America Update, database,” 2011, https:/ /www. itis.gov/ servlet/Sin gleRpt/Sing leRpt? _topic=TS N & search_value=520965#null.

[7] *United States Department of Agriculture (USDA), “*Natural Resources Conservation Service, *PLANTS Database, Amaranthus spinosus access,” 2016,* https://plants.sc.egov.usda.gov/java/.

[8] Pocheon: Korea National Arboretum, *English names for Korean native plants via Korea Forest Service*, pp. 349, 2015.

[9] Flora of China, "*Celosia cristata*," *Missouri Botanical Garden*, St. Louis, MO & Harvard University Herbaria, Cambridge, MA, 2014.

[10] G. A. Pritzel and C. Jessen, “The German folk name of the plants,” *New contribution to the German language treasure*, pp. 86, Philipp Cohen, Hanover. 1882.

S5 Table. Summery of the differences of photosynthetic parameters

	GA-1	GA-2	GrA	TA	RA	CC
	**Field *vs*. GH**	**Field *vs*. GH**	**Field *vs*. GH**	**Field *vs*. GH**	**Field *vs*. GH**	**Field *vs*. GH**
**SPAD**	S	H	H	L	H	L
**LEF**	S	H	L	S	S	H
**ΦII**	L	H	L	H	H	S
**ΦNO**	S	S	S	S	S	S
**ΦNPQ**	S	L	H	S	L	H

S: Significantly similar in field and GH conditions; H: Significantly higher in field compared to GH; L: Significantly lower in field compared to GH. (P<0.05)

S6 Table. Summery of PC analysis for photosynthetic parameters

PC loading status	PC1	PC2	PC3	PC4	PC5
Eigenvalue	2.42	1.54	0.79	0.17	0.07
Proportion of the variance explained	0.49	0.31	0.16	0.04	0.01
Cumulative variance explained	0.49	0.79	0.95	0.99	1.00
**PC representation for each photosynthetic parameter**					
SPAD	-0.48	0.36	0.47	0.64	0.13
LEF	-0.27	-0.59	-0.56	0.51	0.12
ΦII	-0.62	-0.08	-0.01	-0.27	-0.73
ΦNO	0.25	0.61	-0.55	0.33	-0.39
ΦNPQ	0.50	-0.38	0.41	0.40	-0.53
